# Does Europe Need an EU Product Safety Directive for Access Scaffolding?

**DOI:** 10.3390/ijerph16010103

**Published:** 2019-01-02

**Authors:** Juan Carlos Rubio-Romero, Manuel Suárez-Cebador, María del Carmen Pardo-Ferreira, José María de la Varga-Salto, Jesús Antonio Carrillo-Castrillo

**Affiliations:** 1 School of Industrial Engineering, C/ Dr. Ortiz Ramos s/n, Campus de Teatinos, Universidad de Málaga, 29071 Málaga, Spain; juro@uma.es (J.C.R.-R.); suarez_c@uma.es (M.S.-C.); carmenpf@uma.es (M.d.C.P.-F.); jmdelavarga@uma.es (J.M.d.l.V.-S.); 2 School of Industrial Engineering, Universidad de Sevilla, Camino de los Descubrimientos sn, 41092 Sevilla, Spain

**Keywords:** scaffolding, construction sector, occupational health and safety, CE marking, EU product safety directive

## Abstract

The main requirement established for the development of European Union product safety directives is to ensure a high level of safety for users. This research aims to analyze whether Europe needs a product safety directive for scaffolding and identify the main factors to be defined in public policies on the use of standardized scaffolding in the absence of such a directive. The principal types of scaffolding were reviewed, along with European regulations, and their risk levels. Finally, a qualitative study using a panel of experts was conducted to determine the differences between types of scaffolding and whether the enactment of such a directive would be justified. Key results were that the risk level associated with scaffolding positioned it third or fourth between material agents more hazardous in relation to falls from height. There is no existing product safety directive for scaffolding, despite the fact that there are directives for other products less dangerous than or as dangerous as scaffolding. However, there are noncompulsory standards EN 12810-1-2 and EN 12811-1-2-3-4 for scaffolding, which would form the basis of the essential requirements contained in a directive if it were created. The experts highlighted significant differences between “standardized” and “nonstandardized” scaffolding, with higher safety levels and productivity, and better maintenance, inspection, assembly, and dismantling associated with the former, and lower costs with the latter. Thus, they found that the enacting of an EU product safety directive for scaffolding would be justifiable, and in its absence supported the promotion of the use of standardized scaffolding.

## 1. Introduction

Evaluation of the effectiveness of preventive measures is an important challenge in the research field of safety science. In the review of Khazode et al. [1], there are three types of intervention: Engineering, behavioral, and enforcement. In safety science, a large number of studies are dedicated to evaluating interventions. Moreover, any intervention can imply all of the three types. Engineering interventions, such as the replacement of scaffolds, need to be done with behavioral and enforcement interventions.

The benefits of such evaluation research are useful both for practitioners and for public authorities [2].

Falls from height are one of the most significant occupational health and safety problems in the world [3]. In construction, they undoubtedly represent the highest risk. Among the material agents most associated with accidents involving falls from height, access scaffolding or tubular scaffolding is one of the most frequently recorded [4].

In the European Union (EU), the most dangerous industrial products, such as machinery, protective equipment (PPE), and chemical products, are regulated by a specific product safety directive, which sets the minimum essential requirements with which manufacturers must comply to guarantee the highest levels of safety. This is the main reason for enacting product safety directives: Guaranteeing the safety of especially dangerous products. In cases where directives have been enacted, manufacturers may also consult harmonized nonmandatory European standards (EN), which provide details that are not set out in the directive and translate the essential requirements into tangible aspects that help manufacturers with compliance and obtaining a presumption of conformity before mandatory Conformité Européenne (CE) marking [5]. To date, despite the potential risk associated with access scaffolding, there is no specific related product safety directive. However, there are two harmonized nonmandatory (voluntary) standards for access scaffolding: EN 12810 and EN 12811 [6]. These harmonized standards would form the basis of the essential requirements contained in a directive if this hypothetical directive were to be enacted today. The results of a comparative analysis of safety levels between scaffolding that conforms to EN 12810/12811 standards—which we call “standardized scaffolding” or “system scaffolding” —and “nonstandardized scaffolding” would help us to evaluate the benefit of enacting an EU product safety directive for access scaffolding and, therefore, its necessity.

Nowadays, product safety directives are introduced in Europe according to Decision No 768/2008/EC of the European Parliament and of The Council. This Decision indicated that “Community harmonisation legislation shall restrict itself to setting out the essential requirements determining the level of such protection and shall express those requirements in terms of the results to be achieved. Where recourse to essential requirements is not possible or not appropriate, in view of the objective of ensuring the adequate protection of consumers, public health and the environment or other aspects of public interest protection, detailed specifications may be set out in the Community harmonisation legislation concerned. Where Community harmonisation legislation sets out essential requirements, it shall provide for recourse to be had to harmonised standards, adopted in accordance with Directive 98/34/EC, which shall express those requirements in technical terms and which shall, alone or in conjunction with other harmonised standards, provide for the presumption of conformity with those requirements, while maintaining the possibility of setting the level of protection by other means.”

The Decision No 768/2008/EC also states that “Manufacturers shall ensure that the product is accompanied by instructions and safety information in a language which can be easily understood by consumers and other end-users, as determined by the Member State concerned.”

The introduction of a product directive also implies the enforcement by public authorities. For example, Cordero et al. [7] analyzed the efficiency of market control activities in the European framework for machinery.

There is only one previously published analysis [8] that compares the safety levels of scaffolding manufactured according to (European Norm) EN 12810 and EN 12811 with those of nonstandardized scaffolding, and this already reflects the higher levels of safety of the former. The key results of this are summarized in a later section of this paper. The current reality is that these different types of scaffolding continue to coexist on the market. Our study aims to analyze whether Europe needs a product safety directive for access scaffolding, and to identify the main factors to be defined in public policies to contribute, in the absence of such a directive, to encouraging the use of standardized scaffolding rather than other types. To achieve this, we analyzed regulation, occupational safety risk levels, and the advantages and disadvantages of different types of access scaffolding.

This analysis only deals with the technological aspects. Safety depends on other factors, such as work organization, safety management, and worker training and safety culture, among others [2]. However, in this paper, we analyze the differences related to the introduction of a product safety regulation enforcing the use of scaffolding manufactured according to EN 12810 and EN 12811. The proposed regulation would increase safety from a technological point of view but would also implicate the commercialization of scaffoldings with proper instructions for users.

### 1.1. Types of Access Scaffolding

Firstly, the most common types of scaffolding in the European market are presented. They are shown in Table 1. Below, they are described according to their main features. 

Standardized Scaffolding or System Scaffolding. These terms describe scaffolding that conforms to EN 12810 and EN 12811 standards. Currently, the following European standards for scaffoldings are established:

- EN 12810-1. Facade scaffolds made of prefabricated components. Product specifications;
- EN 12810-2. Facade scaffolds made of prefabricated components. Particular methods of structural design;
- EN 12811-1. Temporary works equipment. Scaffolds. Performance requirements and general design;
- EN 12811-2. Temporary works equipment. Information on materials;
- EN 12811-3. Temporary works equipment. Load testing;
- EN 12811-4. Temporary works equipment. Protection fans for scaffolds. Performance requirements and product design.

Standard EN 12810-1 establishes different classes of scaffolding according to different criteria, as can be seen in Table 2.

**Figure 1:**
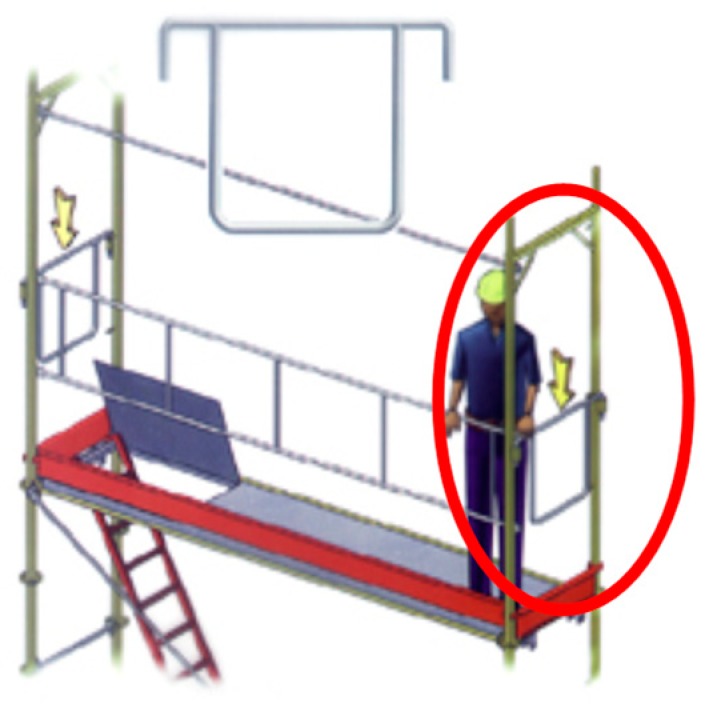
“Standardized scaffolding” or “system scaffolding” (Modular frame scaffolding) [10].

**Figure 2:**
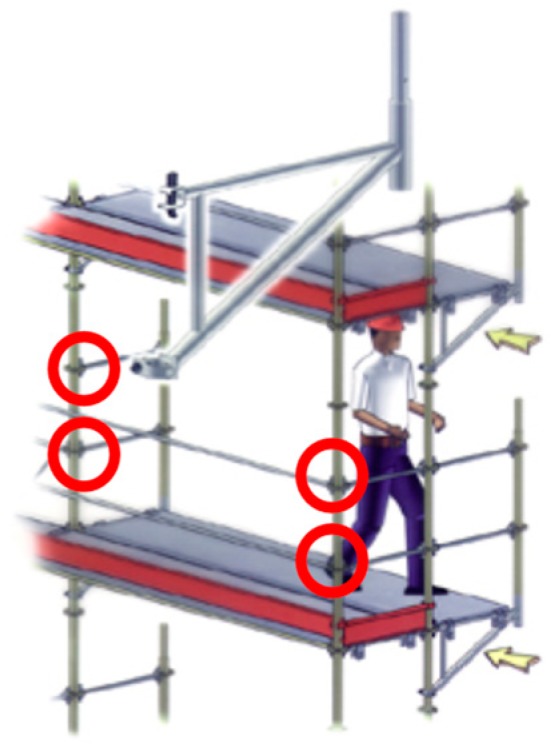
“Standardized scaffolding” or “system scaffolding” (Multidirectional scaffolding) [10].

**Figure 3:**
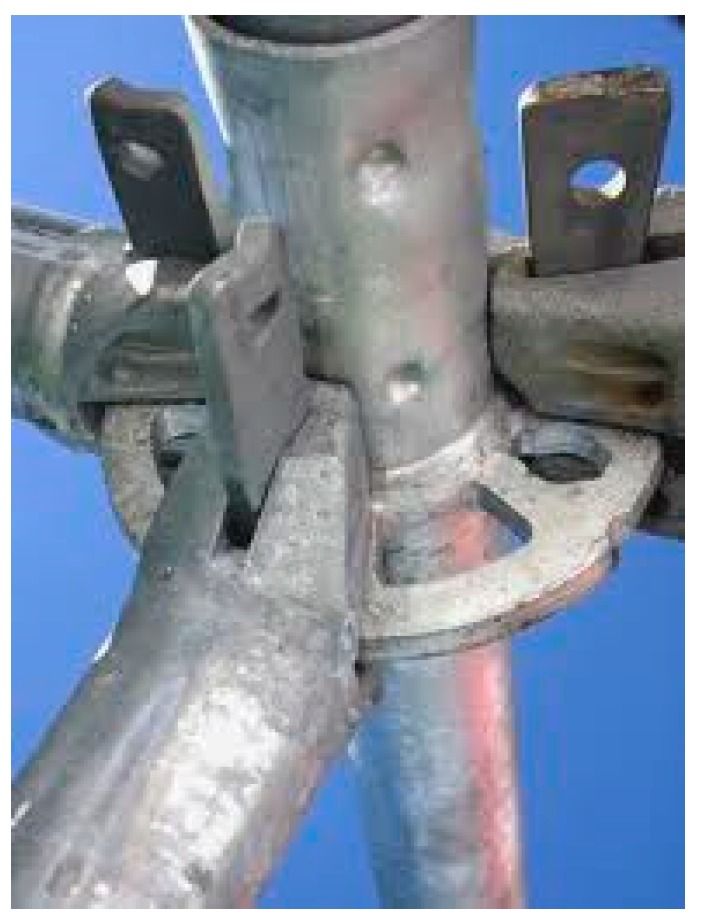
Joining element of multidirectional scaffolding [10].

According to standard EN 12810-2, another classification into two types is established, although both can be combined:

1. Modular frame scaffolding, which can be seen in Figure 1. This scaffolding has standard and transom elements united in a piece called a frame. Obviously, the configurations into which it can be assembled are limited by this circumstance;
2. Multidirectional scaffolding, which can be seen in Figure 2. This scaffolding has various elements separate from each other. This configuration permits different angles, as can be seen in Figure 3.

The main occupational safety features of standardized or system scaffolding are [11]:

- Stairways are mounted on special platforms but not in separated modules;
- Collective protection elements are available on the market for the specific protection of workers who are assembling or dismantling the scaffolds;
- This scaffolding has minimal stability, which makes it necessary to mount the guardrails, with the secondary objective of preventing collapse and thus of increasing occupational safety.

**Figure 4:**
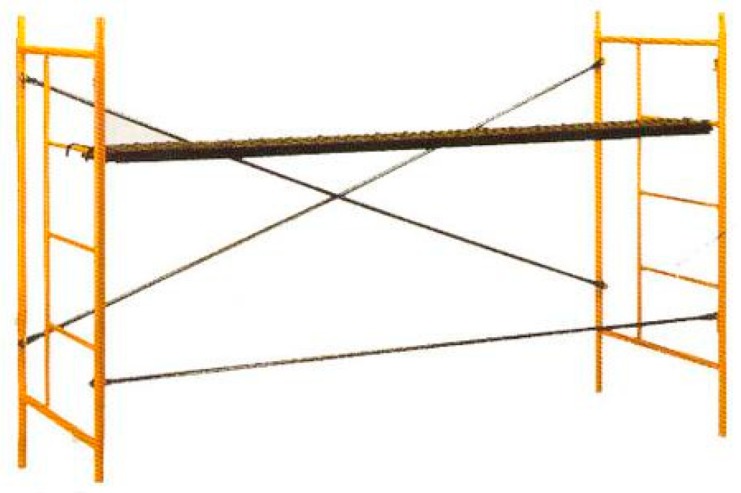
Nonstandardized scaffolding [10].

Nonstandardized Scaffolding. The scaffolding shown in Figure 4 conforms to a private company standard [9].

The main occupational safety features of Spanish nonstandardized scaffolding (Figure 4) are [10]:

- The scaffolding is built using frames. These frames include elements for holding platforms that facilitate the placement of tools and materials, which are not used for mounting ladders or for holding platforms which hold the worker;
- Stairways always require the assembly of a specific module;
- There are no collective protection elements on the market for the specific protection of workers who are assembling or dismantling these scaffolds;
- Good stability due to the St. Andrew’s Cross design makes it unnecessary to mount the guardrails, which have the secondary objective of preventing collapse.

Tube-and-Fittings Scaffolding (Traditional System). “Tube-and-fittings” scaffolding was used in accordance with British Standard BS 5973 until 31 October 2010, and from 1 January 2011 to the present are covered by document TG20: 08 (from the National Access and Scaffolding Confederation), based on the previous BS 5973 standard (now withdrawn) and the EN 12811-1 standard.

**Figure 5:**
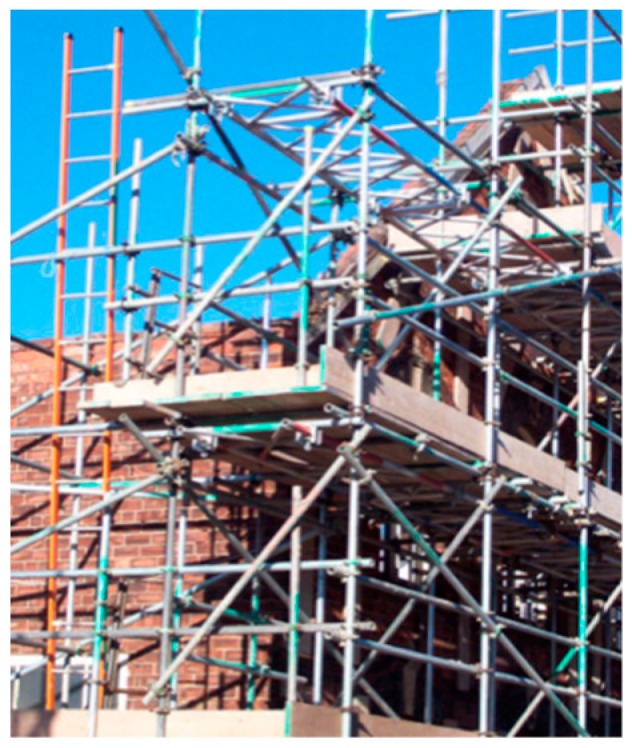
“Tube-and-fittings” scaffolding [10].

The main occupational safety features of tube-and-fittings scaffolding (Figure 5) are [10]:

- Tubes are available in galvanized steel and aluminum; lengths from 1.5 to 5 meters;
- Scaffold boards are available in 2-, 2.5-, 3- and 4-meter lengths. A scaffold board is “banded” on each end with a metal strap to stop the board splitting during misuse and prolonging its serviceable life.
- There are many accessories, such as double couplers for fixing two tubes at 90°, swivel couplers for joining two tubes at an angle, single couplers for joining two tubes in a nonweight loaded position, sleeve couplers for external end-to-end joints, and joint pins for internal end-to-end joints.
- The system provides the flexibility to meet any scaffolding need and system solutions can provide increased speed of assembly.

### 1.2. Risks of Access Scaffolding

Worldwide, falls from height in occupational environments are a major concern [12,13,14], especially in the construction industry [3,15,16]. In the UK, for example, 40 workers per year died from falls from height during the period 2012/2013–2016/2017. This equates to 28% of total occupational fatalities [17]. In the USA, falls accounted for one third of all construction worker fatalities, and for 20% of days off work for construction workers [18]. In Australia, over the eight-year period from 1 July 2003 to 30 June 2011, 232 workers died following a fall from a height. This is 11% of all worker fatalities for the period. This study shows that the construction sector accounted for 37% of fall-related fatalities and recorded a fatality rate four times the average overall rate. Furthermore, it clarifies that falls from scaffolding were the cause of the most time off work [19].

Accordingly, different studies try to quantify risk assessment for falls from height using models such as bowtie [20,21]. In addition, a critical review of the scientific literature related to falls from height in the construction sector shows that the most common factors associated with this kind of occupational accident are high-risk activities, individual characteristics, site conditions, organizational factors, agents (scaffolds/ladders), and weather conditions [4]. 

The British Standards Institute defines “scaffold” as a temporary structure constructed to support a working platform at variable heights [22]. As far as material agents are concerned, scaffolds are a particularly significant source of risk of falls from height on construction sites [4,11,23]. In Spain, the period 2003–2016 gave rise to a total of 56,270 accidents with scaffolds as the material agent associated with deviation, i.e., the “abnormal factor that, alien to usual work activity, brought about the accident”. In this sense, in Spain, scaffolding was one of the material agents most frequently associated with deviation and accounted for one-quarter of construction worker fatalities produced by falls from height [24].

A series of investigations have analyzed safety standards in relation to falls from height. Some studies have statistically dealt with bodily injuries caused as a result of non-occupational, accidental falls (e.g., from ladders and also scaffolding) [25], and there are also epidemiological surveys on the fatality rate of accidental falls (from any kind of structure fixed to buildings in New Zealand), either work-related or not [26].

In the building industry especially, some surveys based on the official accident rates for the construction sector in the USA have examined the injured worker’s profile and the material causative agent involved [27]. In 2009, by means of questionnaires passed to building workers, some researchers also studied the main risk factors in falls from height in connection with different kinds of work equipment [28], and in 2003, Kines [13] studied workers’ behavior in relation to falls from height. Lastly, with the aid of the relevant statistical data on fall-induced fatal accidents on construction sites in the USA, some other surveys have even dealt with the impact of regulatory changes adopted to protect workers against these falls [29].

**Figure 6:**
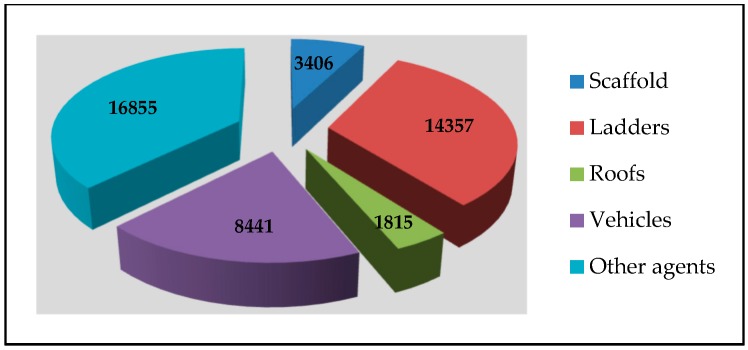
Total injuries due to falls from height in United Kingdom by material agent (2002/03-2007/08) [30].

A report entitled “Statistics of Workplace Fatalities and Injuries. Falls from Height” focused exclusively on falls from height in the UK. Analyzing the data provided in this report, it is possible to determine the precise position of scaffolding in relationship with other material agents. This report presented data for the period 1996/1997 to 2007/2008, particularizing for different material agents and differentiating between fatal and major accidents, and between accidents due to low falls, high falls, and due to falls from height by agent breakdown. Analyzing cumulative total accidents for the period 2002 to 2008, and considering only fatal and major accidents, scaffolding is placed in fourth position behind ladders, vehicles, and plant and earth moving equipment, and ahead of roofs (see Figure 6). Differentiating between fatalities and major accidents, scaffolding places third in the case of fatal accidents, after ladders and roofs, and third in the case of serious accidents, but after ladders and vehicles. Using the same analysis but differentiating between high falls, low falls, and falls due to agent breakdown, again for all types of falls, scaffolding is above roofs and below ladders, vehicles, and plant and earth moving equipment, except in the case of high falls, where scaffolding comes higher than vehicles and plant and earth moving equipment [30].

All these investigations indicate without exception that scaffolding is highly dangerous equipment. According to this information, scaffolding as a product is confirmed as one of the most hazardous agents on the market.

However, we do not have segregated accident statistics to show and measure the difference between standardized/system scaffolding and other types of scaffolding in Europe; not even a study analyzing the different safety levels between all types. Nevertheless, in the USA, Yassin and Martonik [31] estimated the efficiency of the regulatory changes passed in 1996 for the design and assembly of scaffolding. Their study examined the impact of those changes on the accident records and statistical data related to officially detected failures to comply with legal requirements. For a proper estimation, the study compared the relevant records from 1991 to 1996, i.e., prior to the passing of the regulatory changes in 1996, with those from 1997 to 2001. Unfortunately, the study made no distinction between the kind of scaffolding (suspended, mast-climbing, or based on the ground). Even so, it clearly revealed that the new USA standard produced not only a significant decrease in the accident rate but also a reduction in associated costs.

The closest research we have in Spain, which aimed to analyze the safety conditions of supported tubular scaffolds, was carried out in 2007. The authors examined scaffolding erected on 105 building sites in Spain. The study provides a qualitative assessment of the safety conditions of bracings, anchor ties, toe-boards, guardrails, ladders, struts, long beams, cross beams, platforms, supports, etc. The results showed that the general safety level of standardized scaffolding is higher than that of nonstandardized scaffolding, and support EU regulation of the production of this kind of equipment with a specific product safety directive, which could eventually lead to a CE marking on scaffolding and to general implementation of the voluntary standards EN 12810 and EN 12811 among scaffolding manufacturers [8].

### 1.3. European Product Safety Regulations for Access Scaffolding

To guarantee a minimum intrinsic or inherent health and safety level for consumers of a series of high-risk industrial products, such as machinery, PPE, or chemical products, among others [32], prior to their marketing throughout the EU, a number of product safety directives have been issued. Logically, in the workplace, a consumer of machinery, PPE or chemical products is a worker who uses the product. These directives, aside from additional requirements, set the compulsory basic and essential health and safety requirements that the products under these regulations must meet for any manufacturer intending to produce and market them in the EU. As stated in the new approach (NA) and global approach (GA) principles, these compulsory essential requirements are usually developed in the form of voluntary European standards (EN), which provide a detailed description of the technically equivalent conditions to be complied with. These EN standards outline the current interpretation of the essential requirements contained in the corresponding directives. Manufacturers can therefore use the EN standards to design a product fully compliant with the essential requirements of the directives. Declaring that the EN has been duly observed, manufacturers are granted a so-called “presumption of conformity”. The presumption of conformity makes the subsequent compulsory conformity assessment procedure and the CE marking much easier. The whole process, framework, and regulation of the European market cannot be explained in this brief paper without being imprecise. The process is explained in greater detail in References [5,7,33] and, of course, in the regulation framework. 

However, tubular access scaffolding, while considered a very high-risk product, does not have a product safety directive requiring specific compliance from manufacturers [8,10], even though the EU Council stated in its common position on 23 March 2001, with a view to the adoption of an EU directive and to the Council amending Council Directive 89/655/EEC concerning the minimum safety and health requirements for the use of work equipment by workers at work, the intention to take action regarding the requirements for products with the purpose of improving technical features, in particular those of scaffoldings [34].

There is a product safety directive on machinery, which naturally affects mast-climbing and platform lift scaffolding, as they are machines, but there is no product safety directive for tubular access scaffolding. Sometimes, even in the absence of a product safety directive for a particular product, the EU has issued noncompulsory EN standards all the same, outlining the health and safety requirements for the production of that item. This is the case for tubular access scaffolding: Although we do not have a product safety directive for tubular scaffolding, we have two standards for them, EN 12810 and EN 12811, which are updated versions of the previous European harmonization document HD 1000 [34]. We could thus evaluate the safety level of them if a product safety directive for scaffolding were to exist, measuring the safety level of EN 12810/12811 tubular access scaffolding.

### 1.4. European Occupational Health and Safety Regulations on Working Conditions Related to Access Scaffolding

Regarding the use of scaffolding, as with any other work equipment, the occupational health and safety conditions for workers fall under the provisions of the 89/391/EEC Directive, issued by the European Council on 12 June 1989, which concerns measures to be taken to improve employees’ health and safety standards at work [35]. Consequently, work with access scaffolding must currently comply only with 2001/45/EC Directive as issued by the European Parliament and Council on 27 June 2001, which modifies the previous 89/655/EEC Directive, concerning the use of all types of work equipment [36]. This directive essentially regulates the proper way of using all kinds of work equipment but does not regulate the essential physical requirements of the equipment. 

There is no doubt that, for users or workers, one or several product safety directives dedicated to a high-risk product, such as scaffolding, would provide for a more stringent and, consequently, safer standard. In the absence of such directives, health and safety requirements are normally prescribed in the form of the usual work conditions regulations. At the same time, as EU policy on health and safety is becoming more demanding in terms of the publishing of mandatory directives [37,38], to justify a directive, it would be useful to understand the different safety levels between EN 12810/11 standardized scaffolds and other types of scaffolding. 

In Europe, there are specific regulations regarding the working procedures with scaffolds. The Directive 2009/104/EC of the European Parliament and of the Council of 16 September 2009 concerning the minimum safety and health requirements for the use of work equipment by workers at work (second individual Directive within the meaning of Article 16(1) of Directive 89/391/EEC) includes a full section 4.3. in this Directive for “Specific provisions regarding the use of scaffolding”. 

This section includes that “assembly, use and dismantling plan must be drawn up by a competent person,” and that “scaffolding may be assembled, dismantled or significantly altered only under the supervision of a competent person and by workers who have received appropriate and specific training in the operations envisaged, addressing specific risks in accordance with Article 9, and more particularly in:

- understanding of the plan for the assembly, dismantling or alteration of the scaffolding concerned;
- safety during the assembly, dismantling or alteration of the scaffolding concerned;
- measures to prevent the risk of persons or objects falling;
- safety measures in the event of changing weather conditions which could adversely affect the safety of the scaffolding concerned;
- permissible loads;
- any other risks which the abovementioned assembly, dismantling or alteration operations may entail.”

Therefore, the enforcement regulation regarding working procedures and training of workers are already in the European Framework, but the decision to enforce product safety regulation for scaffolds and other construction equipment is not yet approved. We must bear in mind that Directive 2009/104/CE is part of the Occupational Health and Safety Framework (what employers need to comply with) and not of the product safety regulation (what equipment needs to be commercialized).

## 2. Materials and Methods 

A qualitative study of occupational health and safety related to scaffolding in the construction sector was conducted using a panel of 12 Spanish experts, all graduate engineers and with more than seven years of experience in the sector. 

An ad hoc questionnaire was designed for this purpose, with the objective of evaluating different factors related to the level of safety of the four types of scaffolding described above in Table 1. Therefore, in the process of designing the items of the questionnaire, the criteria proposed by the National Institute of Safety and Hygiene at Work of Spain [9] for the classification of the types of scaffolding were used. The stages linked to the use of the scaffolds were determined in accordance with previous research [39,40]. In order to achieve the objective of this study, some questions focused on evaluating the need for the new directive and possible improvements derived from it were included. In addition, other additional aspects that have been explored are those related to productivity/efficiency, costs, and the market. Based on this, the questionnaire was designed and reviewed in conjunction with five different experts, who were not participants in the panel used for the evaluation of scaffolding. Questions were included to determine the greater or lesser prevalence on the market of the different scaffolding systems under analysis, and how they might influence accident rates and occupational safety through their use, efficiency, and costs.

During 2017, based on personal interviews structured around the questionnaire, the various experts participating in the study provided evaluations of the different aspects, and additional comments.

The questionnaire was structured in five parts, as follows:

(A) Estimation of the percentage use of the different types of scaffolding in Spain;

(B) Assessment of risk levels;

B1. Evaluation of safety levels of the different scaffolding systems. This involved ranking the different systems by safety level with a value of 1 to 4, with 1 being the safest and 4 the least safe;

B2. Evaluation of the need for a specific EU product safety directive for access scaffolding. The following question was asked: Do you think that a specific European directive for scaffolding on the health and safety of the product (CE marking) would contribute to improving the occupational health and safety outcomes for workers? A Likert scale (1-5) of possible answers was established with the following options: 1. Definitely YES; 2. Probably YES; 3. I’m not sure; 4. Probably NO, and 5. Definitely NO;

B3. Evaluation of safety improvement through the training of scaffolding assemblers. If a safety directive for scaffolding were enacted, do you think it would be necessary to provide specific training and qualifications for scaffolding assemblers in order to improve the safety levels of scaffolds? A Likert scale (1-5) of possible answers was established with the following options: 1. Definitely YES; 2. Probably YES; 3. I’m not sure; 4. Probably NO, and 5. Definitely NO;

(C) Identification of the strengths and weaknesses of each type of scaffolding in relation to occupational health and safety during the different stages of assembly and use. The experts were asked to indicate the strongest and weakest points regarding safety of each of the scaffolding systems under analysis in each of the following stages of use:

C1. BEFORE assembly;
C2. DURING assembly and dismantling;
C3. AFTER assembly, during use;

(D) Assessment of productivity/efficiency, costs and the market. This implied asking the experts for evaluation rankings of the following:

D1. Level of productivity/efficiency in the different stages of assembly and use. The experts were asked to give the different scaffolding systems a productivity/efficiency ranking of 1 to 4, with 1 representing the highest and 4 the lowest, in each of the following stages:

Loading, unloading, and on-site storage;

- Assembly;
- Inspection;
- Maintenance;
- Use (once assembled);

D2. Cost of acquisition, through either purchase or rental. The experts were asked to rank the different scaffolding systems from the lowest cost (1) to the highest cost (4);

D3. Identification of the factors most influencing the choice of scaffolding system. In this case, the experts were asked to rank the above criteria of productivity and cost by significance in the choice of scaffolding system, from minor importance (1) to greater importance (6):

Loading, unloading, and on-site storage;

- Assembly;
- Inspection;
- Maintenance;
- Use (once assembled);
- Cost of acquisition (purchase or rental).

Parts D1 and D2 would be of interest to promote the adoption of a CE marking according to Decision No 768/2008/EC of the European Parliament and of the Council as any new product harmonization legislation shall restrict itself to setting out the essential requirements determining the level of such protection and shall express those requirements in terms of the results to be achieved.

From the data obtained, a descriptive statistical treatment of the collected information was carried out, determining both the mean and standard deviation. Additionally, following the recommendations of Wilkinson [41] and the American Psychological Association [42], to better visualize the results, classification of the rankings is presented using a 95% confidence interval based on the consensus of the evaluations given by each of the participating experts and calculated based on the mean and standard deviation of the results obtained. 

## 3. Results and Discussion

### 3.1. Estimation of the Percentage Use of the Different Types of Scaffolding in Spain

**Figure 7:**
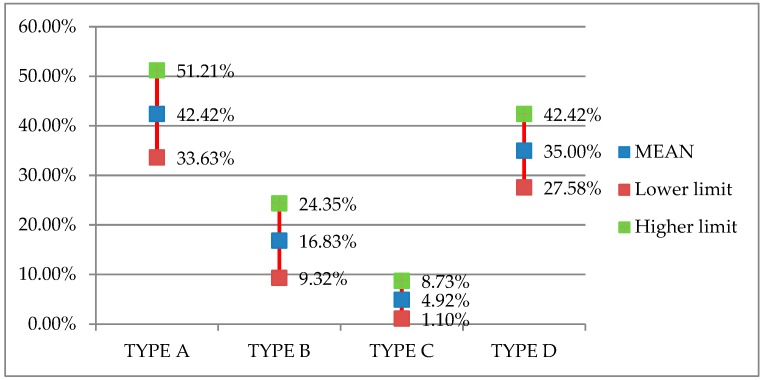
Estimated percentage use of scaffolding types in Spain.

Analysis of the results obtained from the experts’ estimations of the percentage use in Spain of each of the four types of scaffolding systems (see Figure 7) shows that Type A: System scaffolding (frame) is the most used in Spain, with a mean value of 42.42%, and a 95% confidence interval of between 33.63% and 51.21% prevalence among the total scaffolding installed nationally. The next most used system would be Type D: Nonstandardized scaffolding, with an established mean of 35%, and a 95% confidence interval of 27.58–42.42%; followed by Type B: System scaffolding (multidirectional–modular), with a mean of 16.83% and a 95% confidence interval of 9.32–24.35%, and finally Type C: Tube-and-fittings, with a mean of 4.92% and a 95% confidence interval of 1.10–8.73%.

### 3.2. Assessment of Risk Levels

#### 3.2.1. B1. Evaluation of Safety Levels of the Different Scaffolding Systems

The safety level ranking of the different types of scaffolding under analysis is based on values of 1, being the safest, to 4, the least safe. Analysis of the experts’ evaluations is reflected in the results shown in Table 3 below, showing the mean values for the estimated levels of safety and the corresponding 95% confidence intervals. Thus, scaffolding system Type A is identified with a mean value of 1.25 as the safest, followed by Type B (mean value 1.67) and Type C (2.71), with Type D (mean value 3.33) being identified as the least safe. These results are consistent with those obtained by Reference [8] only in relation to scaffolding types A, B, and C, since Type D was not included.

Even though the experts recognize the higher levels of safety in scaffolding types A and B, different studies highlight the need to redesign and integrate new technologies, such as BIM (building information modeling), to incorporate existing scaffolding systems to reduce their complexity so that it would take less effort to assemble and dismantle them, and allow them to be used more reliably to reduce the associated risks [39,43].

#### 3.2.2. B2. Evaluation of the Need for a Specific EU Product Safety Directive for Access Scaffolding

**Figure 8:**
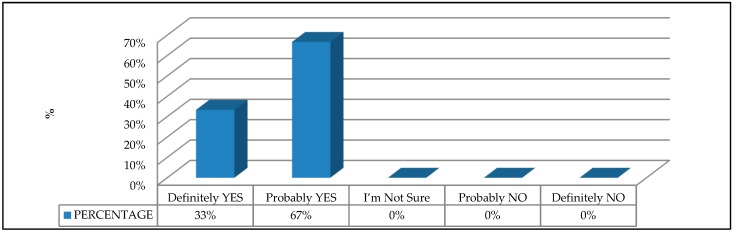
Evaluation of the need for a specific European Union product safety directive for access scaffolding.

The experts agreed that enacting a specific EU product safety directive for scaffolding would be positive, with 67% indicating that it would probably be positive, and 33% indicating that it would definitely be positive (Figure 8). This result is in line with other studies that justify the need to implement other product safety directives—for example, for the design and manufacture of machinery that may pose a risk to workers [8,33,44]. However, the complexity of enacting this directive is recognized in view of the fact that the implementation of a product safety directive implies finding a delicate balance between the market and safety requirements [45].

#### 3.2.3. B3. Evaluation of Safety Improvement through the Training of Scaffolding Assemblers

Given its significance, the experts were also asked whether it would be essential to require specific training for scaffolding assemblers if a scaffolding safety directive were enacted. This question referred to the need for training beyond what is generally required for workers by the 89/391/EEC Directive, thus defining content, duration, and accreditation of the trainers.

**Figure 9:**
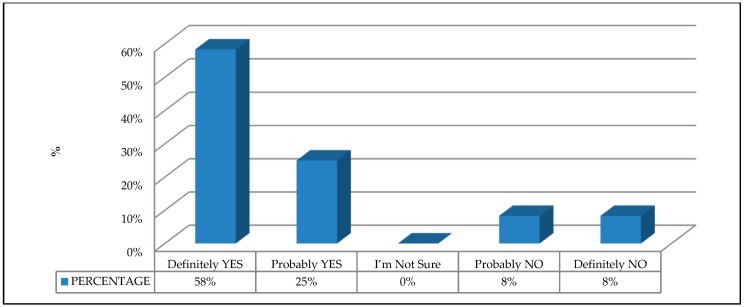
Evaluated need for specific training and qualifications for scaffolding assemblers if enacting an European Union product safety directive for access scaffolding.

Some 84% answered “Probably yes” or “Definitely yes”, as can be seen in Figure 9. According to the results obtained and to other research [46], the need for this specific training and qualification of scaffolding assemblers is clear, to improve safety conditions during the assembly, use, and dismantling of scaffolding, and to ensure the proper supervision, control, and maintenance of scaffolding [44,45,46,47]. Logically, the need to establish this type of specific training is even greater in the case of nonstandardized scaffolding, while its use remains permitted.

### 3.3. Identification of the Strengths and Weaknesses of Each Type of Scaffolding in Relation to Occupational Health and Safety during the Different Stages of Installation and Use

As described in Table 4 below, the analysis carried out summarizes the various strengths and weaknesses related to safety that the participating experts evaluated for each of the different types of scaffolding system under analysis in each of the different stages of use.

According to the results obtained in the analysis of weaknesses and strengths in the various scaffolding systems, differences between each of them are identified according to the stages of use [48,49]. This view coincides with other studies where technical profiles such as Project Manager, Health and Safety Officer, and Site Engineer have confirmed that they agree on the existence of differences in the safety and use of these systems depending on type of scaffolding system, the scaffold material, the procurement of the scaffolding, and sometimes on management aspects related to the scaffolding as it is assembled and used on site [50].

### 3.4. Assessment of Productivity/Efficiency, Costs, and the Market

#### 3.4.1. D1. Level of Productivity/Efficiency in the Different Stages of Assembly and Use

Results of the assessment of productivity and efficiency of the different types of scaffolding in relation to the different activities carried out with them are shown in Table 5 below. The rankings are based on the mean values obtained, with a 95% confidence interval.

#### 3.4.2. D2. Cost of Acquisition, through either Purchase or Rental

The analysis related to the evaluation of the economic cost of the purchase or rental of each of the different types of scaffolding systems is reflected in Table 6 below, where they are ranked in order of cost from lowest to highest. The rankings are based on the mean values obtained, with a 95% confidence interval.

#### 3.4.3. D3. Identification of the Factors most Influencing the Choice of Scaffolding System

The results obtained from the analysis of factors influencing the choice of one scaffolding system over another are shown below in Table 7. As with the other elements of our analysis, rankings are based on the mean values obtained, with a 95% confidence interval. Clearly, the primary factor is the cost of acquisition, followed in order by ease of assembly, productivity in use, efficiency of loading/unloading and storage, maintenance, and finally, efficiency of inspection. 

## 4. Conclusions

A product safety regulation would implicate the assurance of the use of more safe scaffolds in Europe. At the same time, regulation of working procedures incorporated to the instructions would facilitate the standardization of safer use of scaffolds.

There can be no doubt that access scaffolding is one of the most hazardous products used in the workplace. The accident data clearly indicate that scaffolding in general, as a material agent in falls from height, is at the top of the rankings, behind ladders and, depending on the characteristics of the accident, behind vehicles, plant and earth moving equipment, and workplace roofs. In this sense, as a high-risk product, scaffolding deserves to be regulated by an EU product safety directive. Further to this, the evaluated safety levels of standardized scaffolding—i.e., scaffolding that would conform to a hypothetical product safety directive—are significantly higher than for other types of scaffolding, which again justifies regulation by a specific directive. Additionally, experts indicate that the impact of such a directive would be positive.

At the same time, while it has been found that most of the scaffolding used in Spain is standardized or system scaffolding, experts have also identified a significant percentage of nonstandardized scaffolding in use. Since the latter imply lower levels of safety, the relevant authorities should promote mechanisms that prevent their use as far as possible, without prohibiting them as long as there is no specific EU product safety directive, or otherwise encourage the use of standardized scaffolding instead. 

For the establishment of public policies promoting the use of some types of scaffolding over others, this study has identified several relevant factors that could facilitate their design. Thus, cost appears to be the main factor in the continued use of nonstandardized scaffolding, as all types of standardized scaffolding—especially modular frame scaffolding—give better results in terms of the other factors of assembly, dismantling, maintenance, inspection, and even productivity, which could justify the higher cost of acquisition over a medium–long term. These results suggest that the relevant authorities should focus on two types of actions: Firstly, conducting information campaigns to raise awareness of the existing advantages of standardized scaffolding among companies in the sector; and, secondly, making standardized scaffolding more attractive in terms of cost by means of government subsidies or preferential purchase policies. 

One final reason in favor of enacting the type of directive under discussion, which may seem obvious but deserves consideration, is that it would eliminate the need for spending additional energy and resources on promoting the use of scaffolding systems that comply with established standards, since they would be mandatory. 

It is also important to emphasize that, according to the experts, once a product safety directive for scaffolding is enacted, it would be important to simultaneously establish the requisite training and qualifications for scaffolding assemblers, which would be compulsory, in order to guarantee proper assembly and dismantling in conditions of optimum safety.

## Figures and Tables

**Table 1:** Types of scaffolds [9].

TYPE OF SCAFOLDING	
Description	Type
**STANDARDIZED SCAFFOLDING**	
System Scaffolds (Frame). European standard EN 12810/EN12811. The most typical type of scaffold in use on mainland Europe has been the frame system, with its limited number of components and fixed, narrower widths. Faster assembly is possible on the right projects, particularly with mechanical hoisting.	TYPE A 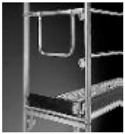
System Scaffolds (Multidirectional–modular). European standard EN 12810/ EN12811. Multidirectional–modular systems with individual prefabricated components, which are more versatile, have begun to grow in popularity.	TYPE B 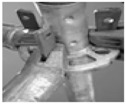
**TRADITIONAL SYSTEMS**	
Tube-and-Fittings (Traditional system). Traditional tube-and-fittings scaffolds have never been widely used outside of the UK, except for very complex applications. They may be found at times in Germany, Italy, and Scandinavia.	TYPE C 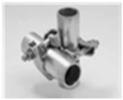
**NONSTANDARDIZED SCAFFOLDING**	
Nonstandardized Scaffolds. Box frame is the simplest scaffolding system. One set of box frame scaffolding includes two box frames, two pairs of cross bars, and four joint pins. In Spain, commonly called “yellow scaffolding”.	TYPE D 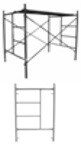

**Table 2:** Different classes of scaffolding according to different criteria set by standard EN 12810-1.

Classification Criteria	Class
Service load	2, 3, 4, 5, and 6, in accordance with EN 12811-1 (see table 3 of this standard)
Platforms and their supports	(D) Drop test, or (N) Nondrop test design
System width	SW06, SW09, SW12, SW15, SW18, SW21, SW24
Headroom	H1 and H2, in accordance with EN 12811-1 (see table 4 of this standard)
Cladding	(B) With or (A) Without cladding facilities
Vertical access method	(LA) Ladder, (ST) stairway, or (LS) both

**Table 3:** Evaluation ranking of safety levels of different types of scaffolding.

Type	Ranking			
	1	2	3	4
A	1.25 (0.99–1.51)			
B		1.67 (1.34–1.99)		
C			2.71 (2.35–3.08)	
D				3.33 (2.96–3.7)

**Table 4:** Strengths and weaknesses of scaffolding systems in the different stages of their use.

		Stages of Use		
Type of Scaffolding	Weaknesses/Strengths	Before Assembly (on site)	During Assembly and Dismantling	After Assembly, During Use
A	STRENGTHS	Standardized	Stability	Easy access
		Adaptable to the needs of the project with prior planning	Specialized assembly	Stability
		Collaboration between assembly and building companies in prior planning and design		Safety
	WEAKNESSES	Requires many parts	Requires anchoring	Modification difficult
		Incompatible with complex facade exits		Narrow
		In many cases an exhaustive prior planning is not done		
B	STRENGTHS	Standardized	Multi-directional	Easy access
		Greater compatibility and flexibility in design	Versatile and flexible	Stability
		Collaboration between assembly and building companies in prior planning and design	Specialized assembly	Safety
	WEAKNESSES	Requires many parts	Requires anchoring	High maintenance
		Very heavy		Narrow
		Complex design		
C	STRENGTHS	Very flexible and adaptable to all needs	Versatile and flexible	Provides access to complex areas
		Fewer parts	Specialized assembly	
		Lighter		
	WEAKNESSES	Nonstandardized	Complicated assembly	Complicated inspection/verification
		Little known or used in Spain	Requires anchoring	High maintenance
		Complex design		
D	STRENGTHS	Cost	Easy assembly	Low maintenance
		Availability	Wider	Freestanding
				Wheels can be used
	WEAKNESSES	Nonstandardized	Heavy parts	Insecure access
		Not adaptable	Nonspecialized assembly	Unstable
			No guardrails or baseboards	Unsafe

**Table 5:** Evaluation ranking of safety levels of different types of scaffolding.

Productivity/Efficiency of	Type	Ranking			
		1	2	3	4
Loading/unloading/on-site storage	A	1.64 (1.16-2.11)			
	B			2.78 (2.06-3.49)	
	C				2.89 (2.20-3.58)
	D		2.18 (1.49-2.87)		
Assembly	A	1.55 (1.24–1.85)			
	B			2.34 (1.77–2.90)	
	C				3.56 (3.21–3.9)
	D		2.27 (1.52–3.02)		
Inspection	A	1.33 (1.05–1.61)			
	B		2.40 (1.97–2.83)		
	C				3.40 (3.08–3.72)
	D			2.50 (1.80–3.20)	
Maintenance	A	1.25 (0.99–1.51)			
	B		2.30 (1.79–2.81)		
	C				3.30 (3.00–3.60)
	D			2.75 (2.11–3.39)	
Use (after assembly)	A	1.46 (1.15–1.76)			
	B		1.78 (1.06–2.49)		
	C			2.70 (1.98–3.42)	
	D				3.09 (2.53–3.65)

**Table 6:** Cost ranking of types of scaffolding systems.

Productivity/Efficiency of	Type	Ranking			
		1	2	3	4
Minimum cost	A		2.60 (2.17–3.03)		
	B				3.13 (2.55–3.70)
	C			3.00 (2.27–3.73)	
	D	1.10 (0.9–1.3)			

**Table 7:** Ranking of factors influencing the choice of scaffolding type.

Productivity/Efficiency of	Ranking					
	1	2	3	4	5	6
Efficiency of loading/unloading/storage				3.67 (2.89–4.44)		
Efficiency of assembly		2.37 (1.82–2.91)				
Efficiency of inspection						4.90 (4.28–5.52)
Maintenance efficiency					4.90 (4.28–5.52)	
Productivity in use			3.40 (2.42–4.38)			
Cost of purchase or rental	1.92 (0.83–3.01)					
